# Abstracts From the Third Annual Research Day Hosted by the Michigan State University College of Osteopathic Medicine, Novi, Michigan, May 13, 2025.

**DOI:** 10.51894/001c.144847

**Published:** 2025-09-30

**Authors:** Francis O. Akenami, Rana Ismail, C. Patricia Obando S., Andrea Amalfitano

**Affiliations:** 1 College of Osteopathic Medicine Michigan State University

## Abstract

The *Spartan Medical Research Journal (SMRJ)* is pleased to feature abstracts from the Third Annual Research Day of the Michigan State University College of Osteopathic Medicine (MSUCOM), held on May 13, 2025, in Novi, Michigan. This event was jointly sponsored by MSUCOM, the Graduate Medical Education Alliance (GME Alliance), and Research, Innovation, and Scholarly Engagement (RISE). A total of 152 research abstracts were selected following a rigorous blinded review conducted by the MSUCOM Research Day Planning Committee in collaboration with the SMRJ editorial team. These abstracts were presented at the Research Day, with awards conferred for outstanding oral and poster presentations. Of the 148 accepted presentations, 132 authors consented to publish their abstracts in *SMRJ*.

The included abstracts span a wide range of contemporary topics in medical and clinical sciences. They employ diverse study designs, including basic science investigations, clinical studies, case reports, medical education projects, and quality improvement initiatives. While these abstracts provide concise summaries of the work presented, they do not allow for a complete assessment of the scientific rigor or generalizability of the findings. Many represent preliminary results that may require further refinement, validation, and eventual full publication. Nonetheless, they play an essential role in disseminating new ideas and fostering dialogue among clinicians, researchers, and educators, thereby advancing the fields of medicine and osteopathic practice.

The MSUCOM Research Day Committee and *SMRJ* strongly encourage authors to pursue full-length manuscript publication to further contribute to the academic community. We thank all contributors and look forward to another outstanding season of submissions for MSUCOM Research Day 2026.

Andrea Amalfitano, DO, PhD

Osteopathic Heritage Foundation Professor of Pediatrics, Microbiology and Molecular Genetics

Professor, BioMolecular Science Gateway

Editor-in-Chief, Spartan Medical Research Journal (SMRJ)

MSU College of Osteopathic Medicine- Graduate Medical Education Alliance

C. Patricia Obando S., PhD.

Associate Dean and DIO, Graduate Medical Education

Associate Professor- MSU College of Osteopathic Medicine- Graduate Medical Education Alliance

Rana Ismail, PhD, MSc, CPHQ

Director of Research

Editor, Spartan Medical Research Journal (SMRJ)

MSU College of Osteopathic Medicine- Graduate Medical Education Alliance

Francis O. Akenami, BMLS, PhD, MSc, FIMLS

Managing Editor

Spartan Medical Research Journal (SMRJ)

MSU College of Osteopathic Medicine- Graduate Medical Education Alliance

